# Impact of Non-Surgical Periodontal Treatment on the Concentration and Level of MRP-8/14 (Calprotectin) as an Inflammatory Biomarker in Women with Periodontitis and Rheumatoid Arthritis: A Quasi-Experimental Study

**DOI:** 10.3390/diseases12010012

**Published:** 2024-01-01

**Authors:** Elena Aurora Popoca-Hernández, Rita Elizabeth Martínez-Martínez, Roberto Fidencio González-Amaro, Perla del Carmen Niño-Moreno, José Luis Ayala-Herrera, Alberto Vinicio Jerezano-Domínguez, Leon Francisco Espinosa-Cristóbal, María de Lourdes Márquez-Corona, Irene Aurora Espinosa-de Santillana, Carlo Eduardo Medina-Solís

**Affiliations:** 1 Doctoral Program in Basic Biomedical Sciences, Faculty of Medicine, Autonomous University of San Luis Potosi, San Luis Potosi 78210, Mexico; elena.popoca@correo.buap.mx; 2 Master Program in Advanced Dentistry, Faculty of Dentistry, Autonomous University of San Luis Potosi, San Luis Potosi 78290, Mexico; 3 Department of Immunology, Faculty of Medicine, Autonomous University of San Luis Potosi, San Luis Potosi 78210, Mexico; rgonzale@uaslp.mx; 4 Center of Research in Health Sciences and Biomedicine, Faculty of Medicine, Autonomous University of San Luis Potosi, San Luis Potosi 78210, Mexico; ncarmenp@uaslp.mx; 5 School of Dentistry, Universidad De La Salle Bajío, Leon 37150, Mexico; jayala@delasalle.edu.mx; 6 School of Stomatology, Meritorious Autonomous University of Puebla, Puebla 72410, Mexico; alberto.jerezano@correo.buap.mx (A.V.J.-D.); irene.espinosa@correo.buap.mx (I.A.E.-d.S.); 7 Master Program in Dental Sciences, Stomatology Department, Institute of Biomedical Sciences, Autonomous University of Juarez City, Ciudad Juárez 32310, Mexico; leon.espinosa@uacj.mx; 8 Academic Area of Dentistry, Health Sciences Institute, Autonomous University of Hidalgo State, Pachuca 42160, Mexico; lmarquez@uaeh.edu.mx (M.d.L.M.-C.); cemedinas@uaeh.edu.mx (C.E.M.-S.)

**Keywords:** oral health, periodontitis, periodontal treatment, inflammatory biomarkers, rheumatoid arthritis

## Abstract

The aim of this study was to evaluate the impact of non-surgical periodontal treatment (NS-PT) on periodontal parameters and inflammatory biomarkers in the concentration and level of calprotectin (CLP) in women with periodontitis and rheumatoid arthritis (RA). In this quasi-experimental study, we evaluated 30 women (mean age: 52.0 ± 5.8 years) with periodontitis and RA who had been diagnosed and treated for RA for more than 3 years and whose activity markers remained at similar values without significant reduction over three consecutive months. Patients underwent NS-PT, which included plaque control, scaling, and root planing. Serum and saliva samples, periodontal indices, RA activity markers, Disease Activity Score-28 (DAS28), the erythrocyte sedimentation rate (ESR), and the C-reactive protein (CRP) and CLP contents were measured at the beginning of the study and 6 and 12 weeks after NS-PT. Parametric and nonparametric tests were used in the analysis. The mean age was 52.0 ± 5.8 years. Compared to the baseline results, all periodontal indices were significantly reduced 6 and 12 weeks after NS-PT (*p* < 0.001). DAS28 was also significantly reduced after 12 weeks (*p* < 0.0001). Similarly, the serum CLP concentration decreased 6 and 12 weeks after NS-PT (*p* < 0.0001). Of the patients, 100% presented lower levels of CRP and ESR (*p* < 0.0001). Overall, NS-PT reduced inflammation and disease activity, highlighting the importance of oral health in the control and treatment of systemic diseases such as RA and confirming that NS-PT effectively reduces periodontitis activity and plays a key role in modulating RA activity. Therefore, NS-PT should be considered as an adjunct treatment for RA.

## 1. Introduction

According to the Global Burden of Disease Study 2017, periodontal disease, a chronic inflammatory disease, is considered the 11th most prevalent condition [1]. It affects tooth-supporting tissues and has effects ranging from gingivitis to the most aggressive forms of periodontitis (PE) that result in tooth loss [2,3]. Following caries, PE is considered the second-most-frequent oral disease. On a global scale, the prevalence of severe periodontal disease has experienced a progressive increase, despite ongoing attempts to prevent and manage the advancement of this condition, and gingivitis and periodontitis now have a global prevalence ranging from 20% to 50% [4]. Periodontal disease affects the tissues that both surround and support the teeth. The disease is characterized by bleeding or swollen gums (gingivitis), pain, and sometimes bad breath. In its more severe form, the gum can come away from the tooth and supporting bone, causing teeth to become loose and sometimes fall out. The main risk factors for periodontal disease are poor oral hygiene and tobacco use. The origin of periodontitis is multifactorial and involves multiple component causes, genetics, epigenetic influences, and modifiable factors such as environmental factors, habits, and medications that establish and propagate periodontitis lesions [5,6]. The appearance of dysbiosis of the subgingival microbiome triggers an inflammatory response. This results in the expression of several mediators that can lead to alveolar bone loss and tooth mobility. In this situation, oral hygiene is essential; however, this is not achieved adequately in the majority of patients, because many of them do not prioritize oral care and hygiene [7,8]. 

The first step in the chronological sequence of periodontal procedures is termed non-surgical periodontal treatment (NS-PT), the cornerstone of periodontal therapy. It includes plaque removal and control, scaling and root planing, and occasionally, the use of chemical agents [9,10]. The goal of NS-PT is to control periodontal infection by removing bacteria and calculus from periodontally involved root surfaces. However, when periodontal tissues require remodeling, surgical periodontal treatment is also indicated. Periodontitis does not only affect dentition; it also represents a threat to general health. Indeed, periodontitis has been associated with different systemic conditions, including adverse pregnancy outcomes [11,12], metabolic syndrome [13], diabetes mellitus [14], osteoporosis [14], cardiovascular disease [15,16,17], Alzheimer’s disease [18], and chronic obstructive pulmonary disease [19]. As a disease in which inflammation is triggered, it has also been associated with the presence of oral, colorectal, or breast cancers [20]. 

In addition, periodontal bacteria and related inflammatory mediators have been demonstrated to modulate systemic inflammatory and autoimmune responses in various diseases, including RA, a chronic inflammatory disease of autoimmune origin [11,12,13,14,15,16,17,18,19,20,21,22,23], and studies have demonstrated a correlation between periodontitis and RA [21,22,23]. RA, a systemic disease, is characterized by the accumulation of an inflammatory infiltrate in the synovial membrane, which leads to the destruction of the joint’s architecture, typically affecting the small joints of the hands and feet, among others, causing losses in function and a decreased quality of life or even death [24,25].The diagnosis of RA and its level of activity are determined using a combination of clinical and laboratory tests, most commonly the Disease Activity Score-28 (DAS28) index, the erythrocyte sedimentation rate (ESR), and the serum level of C-reactive protein (CRP), to establish the baseline values in order to assess the evolution of the disease [26].

Several reports have provided evidence of an association between RA and periodontitis [21,22,23]. Currently, the possible etiopathogenic relationship between RA and PE is being investigated. This is not only limited to the sharing of inflammatory mechanisms but also the importance of periodontal infections due to the role they play in the citrullination of peptides (post-production modification of arginine residues into citrulline) and the associated development of antibodies against citrullinated proteins that are specific to RA and are related to the pathogenesis and severity of the disease. The prevalence of periodontitis is high, and the presence of bacterial DNA from periodontal bacteria has been identified in the synovial fluid, as well as in the serum of patients with RA [27]. The gram-negative bacterium *Porphyromonas gingivalis* is a significant factor in the development of PE and is the only bacterium that expresses the enzyme peptidyl arginine deiminase (PAD), which is responsible for the citrullination process. It has been suggested that infection with this microorganism could accelerate the onset and progression of RA by facilitating the presentation of autoantigens and the expression of autoantibodies against citrullinated peptides (ACPA) that are specific and almost exclusive to RA [26,27,28,29,30].

Some biomarkers common to both diseases have also been detected. However, there is a paucity of information about the effects of periodontal treatment on the evolution and activity level of RA [21,22,27,31]. Common biomarkers of periodontitis and RA activity include calprotectin (CLP), also known as a myeloid-related protein (MRP-8/14) or leukocyte protein L1, which is a heterodimer formed by two calcium-binding proteins S100A8 and S100A9 [27,32]. CLP is predominantly expressed in myeloid cells, such as neutrophils, monocytes, and macrophages [33]. Recently, CLP has been demonstrated to play a role in modulating host adaptive immunity and has been associated with various inflammatory diseases such as RA, systemic lupus erythematosus, Sjögren’s syndrome, pulmonary fibroma, colorectal cancer, and periodontitis [34]. CLP is a highly regulated protein in RA and has been identified in tissues and synovial fluid at sites of active joint destruction produced by macrophages, synovial fibroblasts, and chondrocytes. These cells are involved in the amplification of the inflammatory process, cartilage destruction, and bone resorption [35,36]. In periodontitis, CLP has been identified in crevicular fluid in areas with gingival inflammation. It binds to specific receptors involved in different transduction signals, such as advanced glycation end-products (RAGE) and toll-like 4 receptors (TLR4). These receptors detect pathogen-associated and/or damage-associated molecular patterns (DAMPs) and immediately trigger an inflammatory cascade [37,38]. Their concentrations are correlated with periodontal parameters such as the pocket depth (PD), bleeding on probing (BOP), and gingival index (GI). Notably, human S100A8 is a potent and specific autocrine chemotactic factor in periodontal ligament cells. Accordingly, it may be an attractive therapeutic candidate for the treatment of periodontal disease [39].

Given the association of CLP with RA and periodontitis, the aim of this study was to evaluate the impact of non-surgical periodontal treatment (NS-PT) on periodontal parameters and inflammatory biomarkers in the concentration and level of CLP in women with periodontitis and rheumatoid arthritis (RA).

## 2. Materials and Methods

### 2.1. Study Design 

This quasi-experimental study included 30 female consecutive patients with PE and RA who provided their informed consent. Quasi-experimental study designs are often described as non-randomized, before-and-after, or pre- and post-intervention studies (pretest-posttest). They comprise a broad range of clinical-epidemiological studies in which, for ethical or logistical reasons, it is not possible to carry out randomization or, because the patients need the treatment, a pure experimental study cannot be carried out. They are those studies in which the control and experimental groups comprise the same subjects. Similar to randomized trials, the aim of quasi-experiments aim is to demonstrate causality between an intervention and an outcome. Although the randomized controlled trial is generally considered to have the highest level of credibility with respect to assessing causality, researchers often choose not to randomize the intervention for one or more reasons: (1) ethical considerations, (2) difficulty in randomizing subjects, (3) difficulty in randomizing by locations, and (4) small available sample size [40]. The patients were recruited from the clinics of the Faculty of Dentistry and the Department of Rheumatology of University Hospital of the Meritorious Autonomous University of Puebla, Mexico, in the period from January 2018 to February 2020. Samples were processed as part of the Master’s Laboratory Degree in Dental Sciences from the Autonomous University of San Luis Potosí, San Luis Potosí, SLP, Mexico.

The inclusion criteria were the following: adult women with a minimum of 20 teeth who had been treated with the disease-modifying antirheumatic drug (DMARD) methotrexate for a minimum of 3 years; had at least a 5-year history of RA with similar RA indicator values, without a significant decrease in the last 3 months and rheumatoid factor seropositive and anti-cyclic citrullinated peptide (anti-CCP) antibodies; and had no other diagnosed systemic disease, including Sjögren’s syndrome. Pregnant or lactating women and patients who had undergone prior periodontal treatment were excluded from the study.

### 2.2. Rheumatoid Arthritis Diagnosis and DAS28

The diagnosis of RA was made by an expert rheumatologist, who applied the criteria of the American College of Rheumatology and the European League Against Rheumatism established in 2010 [41]. The DAS28 index was employed to identify the level of disease activity and was performed as previously described in the literature [42]. The RA activity level was considered low, moderate, and high when the DAS28 score was 2.6–3.2, 3.2–5.1, and >5.1, respectively [41].

### 2.3. Periodontal Diagnosis

The diagnosis of periodontitis was made by a single examiner based on criteria previously standardized by an expert periodontist. Patients were diagnosed with generalized chronic periodontitis according to the 1999 Classification of Periodontal Diseases [42]. Patients corresponded to stage II and grade A based on the application of the periodontitis staging and grading system [43].

Other periodontal measurements such as PD and clinical attachment loss (CAL) were performed at six sites per tooth using the standard North Carolina manual periodontal probe (HuFriedy, Chicago, IL, USA) graduated in mm. CAL was calculated by adding the probing depth to the gingival margin level at the recession sites and subtracting the gingival margin level from the probing depth where the margin covered enamel; at the normal level, CAL was coincident with the PD [43]. Periodontal indices, including the oral hygiene index (OHI) [44], GI [45], BOP [46], plaque index (PI) [47], periodontal inflamed surface area (PISA), and periodontal epithelial surface area (PESA) [48], were obtained.

### 2.4. NS-PT

All patients underwent NS-PT. At the first appointment, patients received a verbal explanation about the treatment. A baseline evaluation of the periodontal indices was performed, and blood and saliva samples were collected. Subsequently, patients received oral hygiene instructions and education about the modified Bass technique. Supragingival deposits (plaque and calculus) were then removed using an ultrasonic scaler (Various 350, NSK, Shimohinata, Kanuma, Japan). Patients were instructed to maintain adequate oral hygiene in order to reduce the OHI, PI, BOP, and GI by at least 25% and to be able to continue with the treatment in order to ensure the success of NS-PT during the 12-week study period [49]. At the second appointment, if this reduction had been achieved, patients were treated with full-mouth scaling and root planing (SRP). Patients attended follow-up appointments every 2 weeks. In weeks 6 and 12 post-treatment, samples were recollected, and the indices were re-evaluated. The treatment was carried out on all participants. If, after a week, they did not exhibit reductions of 25%, instructions were given again, and NS-PT was performed until this was achieved. This served as confirmation that the patient already knew the correct techniques to maintain good oral hygiene.

In addition to measurements at the baseline, 6 weeks, and 12 weeks after NS-PT, records were obtained on the day of SRP. “Baseline” refers to the first time the patient arrived and had her periodontal indices checked before the oral hygiene techniques were explained to her and “Day of NS-PT” is the day on which there was already a 25% reduction and the patient underwent NS-PT.

### 2.5. Serum and Saliva Samples

To measure serum concentrations of CLP and CRP, peripheral blood samples were collected from the cubital vein in citrated vacuum tubes. The ESR was determined routinely. Samples were centrifuged at 1500 g for 10 min to obtain the serum [49]. Stimulated saliva samples were collected to measure the CLP concentration. Samples were obtained at the baseline and 6 and 12 weeks after NS-PT. The samples were stored at −40 °C until the performance of enzyme-linked immunosorbent assays (ELISAs) [50].

### 2.6. Enzyme-Linked Immunosorbent Assay

The serum and saliva levels of CLP and CRP were measured using commercial ELISA kits (S100A8/9 Human Elisa Kit 96 test, Invitrogen ThermoFisher, Madison, WI, USA, and CRP Human Instant ELISA Kit 128 test, Invitrogen ThermoFisher, Madison, WI, USA, respectively) according to the manufacturer’s instructions using 50 µL serum or saliva samples. Absorbance was measured and analyzed with a microplate spectrophotometer (Thermo Scientific Multiskan FC Microplate Photometer, Vantaa, Finland).

### 2.7. Statistical Analysis

The examiner was calibrated by an expert in the diagnosis of periodontitis through an intraclass correlation coefficient analysis (k = 0.80). Qualitative data are expressed as frequencies and proportions. Quantitative data are presented as means, standard deviations, and ranges. The Shapiro–Wilk test was performed to determine the normality of the distribution of the variables. Friedman, ANOVA, and Wilcoxon tests were performed to compare the differences between examination periods. The significance level was set to *p* < 0.05. For the data analysis, the statistical program Prism-GraphPad was used.

### 2.8. Ethical Issues

This study was approved by the Research Ethics Committee of the Faculty of Dentistry of the Autonomous University of San Luis Potosi, Mexico (CEI-FE-044-018), and the Research Committee of the Faculty of Dentistry of the Meritorious Autonomous University of Puebla, Mexico (2019110). The study was performed in accordance with the Declaration of Helsinki and the CONSORT guidelines.

## 3. Results

### 3.1. Patients

The interobserver consistency for the results of the 30 patients was >0.80.

### 3.2. Periodontal Findings

The criterion of reductions of at least 25% in the values of the OHI, PI, GI, and BOP was attained (*p* < 0.0001). Significant reductions were observed in all indices 6 and 12 weeks after NS-PT compared to the baseline (*p* < 0.001) (Table 1). No significant differences were noted between 6 and 12 weeks after treatment (*p* > 0.05). A reduction of >50% was observed for PD (4.0, 1.8, and 1.8 mm; *p* < 0.0001). CAL also exhibited a significant reduction among samples (*p* < 0.0001).

The PESA values represent the surface area of the periodontal epithelium, including healthy and inflamed tissues. This index demonstrated a reduction of approximately 70% from the baseline to 6–12 weeks after NS-PT (556.4, 174.1, and 161.1 mm^2^; *p* < 0.0001). The PISA values, which correspond only to the inflamed surface area, exhibited a decrease of >95% from the baseline measurements (352 mm^2^) to 6–12 weeks after NS-PT (16.5 mm^2^) (*p* < 0.0001).

The OHI and dental PI values were reduced from 1.2 ± 0.15 and 2.1 ± 0.09 to 0.3 ± 0.04 and 0.5 ± 0.03, respectively, on the day of the SRP (*p* < 0.0001). These values were maintained at similar levels 6 and 12 weeks after NS-PT (*p* > 0.05) (Table 2).

GI and BOP decreased from 2.8 ± 0.17 and 3.8 ± 0.19 to 1.2 ± 0.1 and 0.8 ± 0.09 on the day of the SRP, respectively (*p* < 0.0001). These two indices were significantly lower 6 and 12 weeks post-NS-PT (*p* < 0.0001). The GI values obtained 6 and 12 weeks after NS-PT were 0.8 ± 0.35 and 0.6 ± 0.27, respectively. The BOP values were 0.5 ± 0.20 and 0.4 ± 0.22 6 and 12 weeks after NS-PT, respectively (*p* > 0.05) (Table 2).

### 3.3. RA Activity Level

**Figure 1:**
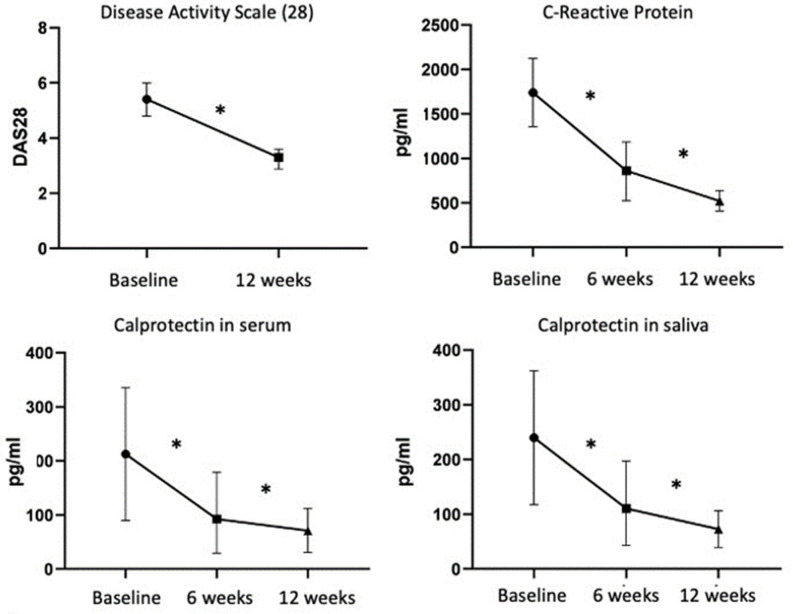
Comparison of AR activity and DAS-28 before treatment and 12 weeks after NS-PT using a Wilcoxon test (* *p* < 0.0001). C-reactive protein (CRP) levels were measured in pg/mL. Values at baseline and 6 and 12 weeks after NS-PT were compared using a repeated-measures ANOVA (* *p* < 0.0001). A comparison of calprotectin in serum and saliva (in pg/mL) at baseline and 6 and 12 weeks after NS-PT was conducted using the Friedman test (* *p* < 0.0001).

The mean ESR values at the baseline and 12 weeks after NS-PT were significantly different (70.5 mm vs. 42.3 mm; *p* < 0.0001), reaching almost-normal values over this period. The DAS28 scores exhibited a significant decrease from 5.4 ± 0.47 at the baseline to 3.2 ± 0.28 12 weeks post-treatment (*p* < 0.0001), representing a decline of >40%. The level of RA activity at the baseline was high in 61.5% and moderate in 38.5% of patients. None of the patients exhibited low levels of RA activity. This situation improved 12 weeks after NS-PT with 57.7% of patients exhibiting reduced RA activity and reaching a moderate level, and 42.3% reaching a low level (*p* < 0.0001). No patient demonstrated a high level of activity post-treatment. Of the patients, 100% exhibited a reduction in the RA activity level, and only one patient demonstrated a reduction from high to low (Table 3, Figure 1).

### 3.4. CLP and CRP

The serum concentration of CRP demonstrated a significant decrease 6 weeks after NS-PT relative to the value at the baseline (861.9 ± 200.1 pg/mL vs. 1739 ± 383.1 pg/mL; *p* < 0.0001). This reduction persisted up to 12 weeks post-intervention, reaching a value of 521.7 ± 114.9 pg/mL (*p* < 0.0001) (Table 4).

Serum CLP was significantly different between the baseline and 6-week post-treatment time points (234.4 ± 113.4 pg/mL vs. 105.6 ± 49.6 pg/mL; *p* < 0.0001) and exhibited a continuous reduction up to 12 weeks after treatment (70.6 pg/mL; *p* < 0.0001). These results are similar to those observed in saliva (baseline: 234.4 pg/mL, 6 weeks: 105.6 ± 49.6 pg/mL, and 12 weeks: 70.6 ± 113.4 pg/mL; *p* < 0.0001). The results obtained for serum were similar to those observed for saliva for the three different samples obtained (*p* < 0.0001).

## 4. Discussion

This study investigated the effects of NS-PT as a treatment for RA. In accordance with our hypothesis, decreases in RA activity (DAS28), serum and saliva concentrations of CLP, ESR, serum CRP levels, and periodontal indices of patients with RA were measured 6–12 weeks post-treatment. A large number of patients were examined in the present study, but only 30 met the inclusion criteria and were included in the final analysis. In addition, some other patients also received NS-PT because they had PE and it was important to perform the treatment; however, they could not be included in the study. In this quasi-experimental study, a control group was not included in accordance with ethical recommendations, and each patient was used as their own control. To proceed with NS-PT, a 25% reduction in periodontal indices was required, as carried out by Walter, since this ensured that the patients were taking care of their oral hygiene. Patients who did not reduce their indices by 25% were taught the techniques again and were evaluated again; i.e., they all received the treatment until they reached a 25% reduction. Only females were evaluated in the study, since, in the biology of autoimmune disorders, it is generally accepted that females constitute a large majority of patients. Autoimmune disorders are characterized by a condition in which the host’s immune system attacks itself by mistake. The mechanism of autoimmune diseases is not clear; however, they present a clear sex bias with a greater prevalence (2 to 1) among females. We assume that the results could also be beneficial for males [50].

Periodontitis is an inflammatory disease with a high global prevalence [7]. Periodontal bacteria and related inflammatory mediators have been demonstrated to modulate systemic inflammatory and autoimmune responses in various diseases, including RA [11,12,13,14,15,16,17,18,19,20,21,22]. Several reports have demonstrated an association between periodontitis and RA activity levels [14,20,21,22,23,24,25,26,27]. NS-PT is performed to eliminate the presence of periodontal bacteria and other local factors such as calculus, which trigger an inflammatory reaction. This procedure involves the control of dental plaque, oral hygiene instructions, and SRP [10]. 

In the current study, significant decreases were observed in periodontal indices (Table 1 and Table 2), highlighting the effectiveness of NS-PT for reducing gingival inflammation. This is in accordance with previous reports demonstrating similar reductions in PD at 3 and 6 months to almost-normal levels [51,52]. Gonzalez et al. conducted a study in which 99 female patients (47.8 ± 15.5 years) were treated with synthetic disease-modifying antirheumatic drugs, and evaluations were performed 3-, 6-, and 12-months post-treatment. A total of 85% of the patients had evaluations 3 months, 58% 6 months, and 53% one year post-treatment. During a 3-month follow-up period, significant decreases were observed in DAS28 scores and the clinical activity of the disease compared to the values at the baseline visit (*p* < 0.05). We used this study as a guide to estimate the results 12 weeks after the treatment [53].

The PESA and PISA indices provide quantitative measures of the inflamed periodontal tissue (mm^2^). In the current study, a reduction of approximately 70% in the PESA and a decrease of >95% in the PISA were observed from the baseline to 6–12 weeks after NS-PT. A significant reduction in the PISA index clearly demonstrates a reduction in the inflammatory component that impacts the levels of systemic biomarkers and response to pharmacological treatment of RA [54]. 

Measurement of the DAS28 index demonstrated a clinical decrease in the RA activity level after NS-PT. This index is considered the gold standard in the clinical evaluation of RA activity. We observed a significant reduction 12 weeks after NS-PT, in agreement with previous studies that reported a significant decrease in DAS28 3 months after NS-PT that remained constant at 6 months compared to an untreated control group [25,53,55]. Before treatment, most of the patients had a high level of RA activity and none of them demonstrated a low level. However, this changed drastically 12 weeks after treatment. During this period, all patients exhibited a decrease in the DAS28 index by one level, and one patient also exhibited a decrease by two levels (from high to low). Notably, none of the patients continued to exhibit high clinical levels of RA activity. This coincides with the report of Cosgarea et al. [52], in which NS-PT was performed on 18 patients with moderate-to-high RA activity (DAS28 ≥ 3.2). After 6 months, RA activity was reduced from moderate to remission (DAS28 < 2.6).

ESR is a laboratory test that reflects inflammatory processes and is used to identify increases in the plasma concentrations of two types of proteins, globulins and fibrinogen, which promote the more rapid sedimentation of erythrocytes during inflammation [56]. This assessment is necessary to determine the DAS28 index. In the present study, we observed a significant reduction at 3 months after NS-PT, in agreement with the results of Zhao et al. [57], who observed decreases in RA indicators (ESR, CRP, and DAS28 index) 3 months after NS-PT.

CRP is a protein produced by the liver that is secreted into the bloodstream within a few hours of the onset of an inflammatory process. Measurement of CRP is a useful tool to monitor the activity of chronic inflammatory diseases such as RA and periodontitis. In our study, NS-PT resulted in a decrease in this biomarker, similar to other studies presented in the literature [53,54,55].

The CLP biomarker is considered a key acute-phase protein that is produced by activated polymorphonuclear leukocytes (PMNs) in vascular tissue and inflammatory lesions. It is relatively stable and easy to measure [33]. CLP is found primarily in human plasma, urine, cerebrospinal fluid, feces, synovial fluid, gingival crevicular fluid, and saliva. It plays major roles in diverse biological functions, such as cell proliferation and differentiation, immunoregulation, oncogenesis, and apoptosis [58]. CLP levels increase significantly in individuals with various infectious and inflammatory diseases, such as pneumonia, septicemia, urinary tract infections, inflammatory bowel diseases (mainly Crohn’s disease and ulcerative colitis), and RA [59]. Elevated CLP levels have been observed in the synovial fluid and plasma of patients with RA compared to healthy individuals. Notably, this outcome is closely associated with the level of disease activity [7]. CLP has recently been considered a key endogenous danger signal or DAMP and is an excellent biomarker for inflammatory processes that trigger intracellular signaling cascades in adjacent cells, thereby inducing inflammatory processes and the release of essential mediators [34]. In the current study, CLP concentrations in both the serum and saliva decreased significantly post-treatment. A search of the literature failed to reveal a similar study that measured the impact of NS-PT on CLP levels in the serum and saliva of patients with RA. Zhou et al. [60] investigated the levels of inflammatory biomarkers including CLP in saliva in individuals with experimental plaque-induced gingivitis. Their results demonstrated that CLP levels in saliva gradually increased and reached a peak on day 21, indicating that CLP levels in saliva may reflect the degree of gingival inflammation. Other reports have demonstrated significant differences in the salivary CLP concentration between periodontally healthy individuals and those with periodontal disease [61]. A study of 969 patients with RA by Jarlborg et al. demonstrated an association between CLP levels and increased RA activity and severity measured using the swollen joint count, DAS, evaluation of health questionnaire joint radiographs, and the ultrasound power Doppler score (USPD) for RA [62].

In terms of limitations, this study was quasi-experimental, only one study was evaluated before, during, and twice after the treatment, and there was no control group. Additionally, the sample size was small. This was because patients had to have a weekly follow-up for 3 months; unfortunately, during this process, the COVID-19 pandemic began and it was no longer possible to continue with the follow-up of the other patients who had begun treatment. It is also likely that the Hawthorne effect occurred; this is a type of human behavioral reactivity in which individuals modify an aspect of their behavior in response to their awareness of being observed. This provides us with guidelines by which to continue the study and expand the sample to verify the results and observe the impact of this treatment in a larger population and for a longer time.

## 5. Conclusions

This study demonstrates that NS-PT is associated with a decrease in RA activity levels based on a significant decrease in the main clinical criterion of RA activity (DAS28 index) and several parameters such as CRP and ESR. NS-PT was also associated with reductions in serum and saliva concentrations of CLP, a key biomarker of RA activity. Collectively, these findings suggest that periodontal health is essential for the adequate control of RA activity. Rheumatologists may consider NS-PT as a tool to provide more effective rheumatologic treatment. Crucially, our study underscores the need to educate patients and professionals about the importance of oral health for their general well-being.

## Figures and Tables

**Table 1:** Periodontal evaluation before and after non-surgical periodontal treatment (NS-PT).

	Baseline Mean ± SD (Range)	6 Weeks after NS-PT Mean ± SD (Range)	12 Weeks after NS-PT Mean ± SD (Range)	*p* Value
PD (mm)	4.0 ± 0.11 (3.7–4.2)	1.8 ± 0.12 (1.7–2.1)	1.8 ± 0.1 (1.6–1.9)	<0.0001 *
CAL (mm)	2.6 ± 0.14 (2.4–3.1)	2.0 ± 0.23 (1.8–2.8)	1.7 ± 0.08 (1.6–1.8)	<0.0001 *
PESA (mm^2^)	556.4 ± 28.8 (487.6–597.0)	174.1± 14.8 (156.6–208.8)	161.1 ± 9.1 (150.5–183.5)	<0.0001 **
PISA (mm^2^)	351.1 ± 29.7 (292.5–398.3)	16.5 ± 6.7 (6.3–28.9)	16.5 ± 6.7 (5.5–29.9)	<0.0001 **

SD: standard deviation, PD: pocket depth, CAL: clinical attachment loss, PESA: periodontal epithelial surface area, PISA: periodontal inflamed surface area. * Friedman test, ** ANOVA repeated measurements. n = 30. The PD, CAL, PESA, and PISA results were measured at the baseline and 6 and 12 weeks after NS-PT. Data are presented as the mean ± standard deviation. PD and CAL were compared using the Friedman test (*p* < 0.0001). PESA and PISA were analyzed using repeated-measures ANOVA (*p* < 0.0001).

**Table 2:** Periodontal indexes at different time points.

	Baseline Mean ± SD (Range)	At Scaling and Root Planing Mean ± SD (Range)	6 Weeks after NS-PT Mean ± SD (Range)	12 Weeks after NS-PT Mean ± SD (Range)	*p* Value *
OHI	1.2 ± 0.15 (1.0–1.6)	0.3 ± 0.04 (0.2–0.4)	0.3 ± 0.04 (0.2–0.4)	0.2 ± 0.04 (0.2–0.3)	<0.0001
PI	2.1 ± 0.09 (1.8–2.2)	0.5 ± 0.03 (0.4–0.5)	0.5 ± 0.03 (0.4–0.5)	0.4 ± 0.05 (0.3–0.5)	<0.0001
GI	2.8 ± 0.17 (2.5–3.0)	1.2 ± 0.1 (1.0–1.5) **	0.8 ± 0.35 (0.2–1.3)	0.6 ± 0.27 (0.0–1.0)	<0.0001
BOP	3.8 ± 0.19 (3.5–4.0)	0.8 ± 0.09 (0.7–1.0) **	0.5 ± 0.20 (0.2–0.8)	0.4 ± 0.22 (0.0–0.8)	<0.0001

SD: standard deviation, OHI: oral hygiene index, PI: plaque index, GI: gingival index, BOP: bleeding on probing. * Friedman test, ** *p* < 0.0001 Wilcoxon test. n = 30. The results of the periodontal indices, OHI, GI, and BOP were measured and compared at four different timepoints (baseline, day of NS-PT, and 6 weeks and 12 weeks after NS-PT). Data are presented as the mean ± standard deviation. The analysis was performed using the Friedman test (*p* < 0.0001). Wilcoxon testing was used to analyze the GI and BOP on the day of the NS-PT and 6 weeks post-treatment.

**Table 3:** Disease Activity Score-28 (DAS28) and erythrocyte sedimentation rate (ESR).

	Before Treatment Mean ± SD (Range)	12 Weeks after NS-PT Mean ± SD (Range)	*p* Value
ESR (mm per hour)	70.5 ± 8.01 (60–82)	42.3 ± 4.81 (36–49.2)	<0.0001 *
DAS28	5.4 ± 0.47 (4.8–6.0)	3.2 ± 0.28 (2.8–3.6)	<0.0001 *
	**Frequency (%)**	**Frequency (%)**	
RA activity level			
Low (DAS28: 2.6–3.2)	0 (0)	11 (42.3)	
Moderate (DAS28: 3.2–5.1)	10 (38.5)	15 (57.7)	< 0.0001 **
High (DAS28: 5.1–8.4)	16 (61.5)	0 (0)	

NS-PT: non-surgical periodontal treatment, SD: standard deviation, RA: rheumatoid arthritis. * Wilcoxon test, ** Fisher’s exact test. AR activity level, comparison of DAS28 and ESR measured at baseline and 12 weeks after NS-PT. Data were analyzed using a Wilcoxon test (*p* < 0.0001). RA activity was divided into low, moderate, and high activity levels based on DAS28 scores. Comparisons were performed using Fisher’s exact test (*p* < 0.0001).

**Table 4:** Inflammatory biomarkers before and after NS-PT.

	Baseline Mean ± SD (Range)	6 Weeks after NS-PT Mean ± SD (Range)	12 Weeks after NS-PT Mean ± SD (Range)	*p* Value
C-reactive protein	1739 ± 383.1	861.9 ± 200.1 ^††^	521.7 ± 114.9	
	(1106–2298)	(522.8–663.4)	(331.7–689.5)	<0.0001 *
Serum calprotectin	205.0 ± 114.0	87.5 ± 48.6 ^††^	66.8 ± 38.1	
	(90.5–391.1)	(29.3–178.9)	(18.5–141.2)	<0.0001 **
Saliva calprotectin	234.4 ± 113.4	105.6 ± 49.6 ^††^	70.6 ± 32.4	
	(107.3–411.9)	(43.0–197.0)	(20.6–120.5)	<0.0001 **
Calprotectin serum vs. saliva	0.3564 ^†^	0.1881 ^†^	0.7059 ^†^	

SD: standard deviation, results expressed in pg/mL. * Repeated-measures ANOVA, ** Friedman test, ^†^ Mann–Whitney U test, ^††^ Wilcoxon test. n = 30. A comparison of inflammatory CRP was performed using a repeated-measures ANOVA (*p* < 0.0001). Serum and saliva CLP levels at baseline and at 6 weeks and 12 weeks were compared using the Friedman test (*p* < 0.0001). A comparison of calprotectin results in serum and saliva was performed using the Mann–Whitney test. All baseline and 6-week results were compared using the Wilcoxon test.

## Data Availability

The datasets generated and/or analyzed during the current study are available from the corresponding author upon reasonable request.
